# Brainstem and Spinal Cord Circuitry Regulating REM Sleep and Muscle Atonia

**DOI:** 10.1371/journal.pone.0024998

**Published:** 2011-10-17

**Authors:** Martina Krenzer, Christelle Anaclet, Ramalingam Vetrivelan, Nishang Wang, Linh Vong, Bradford B. Lowell, Patrick M. Fuller, Jun Lu

**Affiliations:** 1 Division of Sleep Medicine, Department of Neurology, Beth Israel Deaconess Medical Center, Boston, Massachusetts, United States of America; 2 Department of Medicine, Beth Israel Deaconess Medical Center, Boston, Massachusetts, United States of America; 3 Department of Neurology, Philipps-Universität Marburg, Marburg, Germany; Vanderbilt University, United States of America

## Abstract

**Background:**

Previous work has suggested, but not demonstrated directly, a critical role for both glutamatergic and GABAergic neurons of the pontine tegmentum in the regulation of rapid eye movement (REM) sleep.

**Methodology/Principal Findings:**

To determine the *in vivo* roles of these fast-acting neurotransmitters in putative REM pontine circuits, we injected an adeno-associated viral vector expressing Cre recombinase (AAV-Cre) into mice harboring lox-P modified alleles of either the vesicular glutamate transporter 2 (VGLUT2) or vesicular GABA-glycine transporter (VGAT) genes. Our results show that glutamatergic neurons of the sublaterodorsal nucleus (SLD) and glycinergic/GABAergic interneurons of the spinal ventral horn contribute to REM atonia, whereas a separate population of glutamatergic neurons in the caudal laterodorsal tegmental nucleus (cLDT) and SLD are important for REM sleep generation. Our results further suggest that presynaptic GABA release in the cLDT-SLD, ventrolateral periaqueductal gray matter (vlPAG) and lateral pontine tegmentum (LPT) are not critically involved in REM sleep control.

**Conclusions/Significance:**

These findings reveal the critical and divergent *in vivo* role of pontine glutamate and spinal cord GABA/glycine in the regulation of REM sleep and atonia and suggest a possible etiological basis for REM sleep behavior disorder (RBD).

## Introduction

Rapid eye movement (REM) sleep is a distinct behavioral state characterized by an activated cortical and hippocampal electroencephalogram and concurrent muscle atonia. Jouvet and colleagues [1] were the first to identify the pontomedullary junction as a region critically involved in the generation of REM sleep. Later work by Siegel and colleagues showed that the REM-sleep generating neurons were located in a restricted region of the pons [2]. Over the last two decades, research by many, but in particular, Sakai and colleagues [3] has revealed a “REM–on” cell population in the peri-locus coeruleus (peri-LC) alpha in the cat. Boissard and colleagues [4] have recently identified the homologous structure of the cat peri-LC alpha in rats and named this region the sublaterodorsal tegmental nucleus (SLD) as it is located just ventral to the caudal laterodorsal tegmental nucleus (cLDT) and rostral to the LC. Unlike the cat peri-LC-alpha, however, the rat SLD does not contain cholinergic or monoaminergic neurons. Cell-body specific lesions that are restricted to the SLD produced REM without atonia in the rat [5], a phenotype that is similar in appearance to human REM sleep behavior disorder (RBD). Combined cell-body specific lesions of the SLD and cLDT in the rat reduced REM sleep by ca. 60% and, also, produced fragmentation of the REM sleep state [5].

With respect to “REM-off” (inhibitory) neurons, we have previously demonstrated that the ventrolateral periaqueductal gray matter (vlPAG) and lateral pontine tegmentum (LPT) contain a population of GABAergic neurons that project to and inhibit the SLD [5], [6]. Consistent with their putative “REM-off” role, injection of the GABA agonist muscimol into the vlPAG-LPT triggers large amounts of REM sleep in rats [7], [8], cats [9] and guinea pigs [10] while pretreatment with bicuculline blocks the REM-inducing effects of muscimol in guinea pigs [10]. Cell-body specific lesions of the vlPAG-LPT doubled total REM sleep time and increased the number and duration of bouts of REM sleep [5], [11].

We hypothesized that mutually inhibitory REM-off and REM-on areas in the mesopontine tegmentum may form the substrate of the “switching circuitry” for REM sleep. In this putative switching arrangement, GABAergic REM-on neurons of the SLD inhibit GABAergic REM-off neurons of the vlPAG-LPT and *vice versa*. We further hypothesized that descending glutamatergic SLD neurons and glycinergic/GABAergic premotor neurons of the spinal ventral horn play a role in REM atonia. Because, however, glutamate and GABA are intermingled in the SLD, LPT and spinal ventral horn, it has been difficult to characterize the respective *in vivo* roles of these fast neurotransmitters in regulating REM sleep phenomena. To overcome this problem, we used transgenic mice harboring lox-P modified alleles of either the vesicular glutamate transporter 2 (VGLUT2, encoded by *Vglut2*) or vesicular GABA transporter (VGAT, encoded by *Vgat*) genes. By stereotaxically injecting an adeno-associated virus (AAV) vector containing the gene for Cre recombinase (AAV-Cre) into the SLD-cLDT or LPT of *Vglut2* ^flox/flox^ and *Vgat* ^flox/flox^ mice, we selectively and focally eliminated glutamate or GABA/glycine neurotransmission, respectively, in these regions.

## Results

### Experiment 1: Delineating the neuroanatomical SLD and cLDT in the mouse

**Figure 1:**
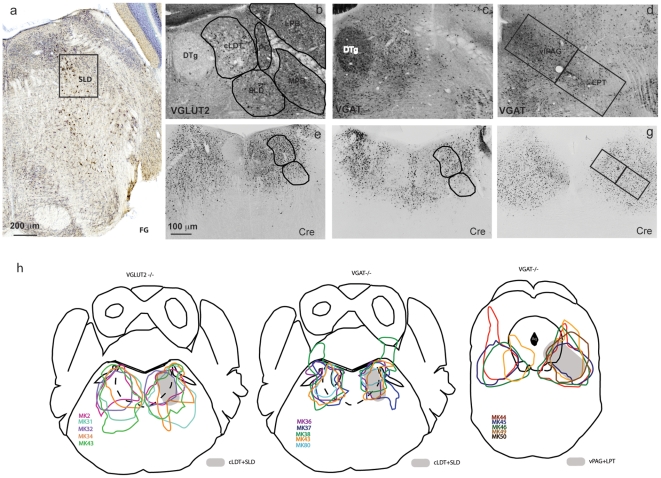
Distribution of VGLUT2-, VGAT- and Cre-positive neurons in the pontine tegmentum. (a) Retrograde-labeled neurons from the spinal cord mark the mouse SLD. VGLUT2-positive neurons labeled by green fluorescent protein (GFP) are seen primarily in the cLDT-SLD, precoeruleus (PC) and parabrachial nuclei (PB) (b), whereas VGAT-positive neurons (also a GFP proxy) are seen primarily in the cLDT and dorsal tegmental nucleus (DTg) but not in the PB-PC (c). VGAT is also highly expressed in the vlPAG-LPT (d). Typical Cre labeled patterns after the AAV-Cre injections targeting the cLDT-SLD are shown in (e) for VGLUT2 deletion and (f–g) for VGAT deletion. The AAV-cre filled areas from each VGLUT2 and VGAT deletion cases are outlined in (h).

As described above, previous work has suggested a critical role for the SLD in the regulation of REM sleep and motor atonia. Because, however, none of this previous work was performed in the mouse, the first necessary step in the present study was to delineate the neuroanatomic locus of the SLD in the mouse. Following the injection of the retrograde tracer Fluoro-Gold (FG) into the mouse ventral spinal horn (see methods), a population of retrogradely labeled FG-positive neurons was identified in the mesopontine tegmentum, just ventral to the region corresponding to the mouse cLDT (Figure 1a). The locations of SLD and cLDT are therefore similar to those in the rat [5], [12] and this FG-labeled population formed the basis of SLD coordinates for AAV-Cre or AAV-GFP injections in the remaining experiments (Figure 1a).

### Experiment 2: Determining the role of cLDT-SLD glutamate in REM sleep regulation

**Figure 2:**
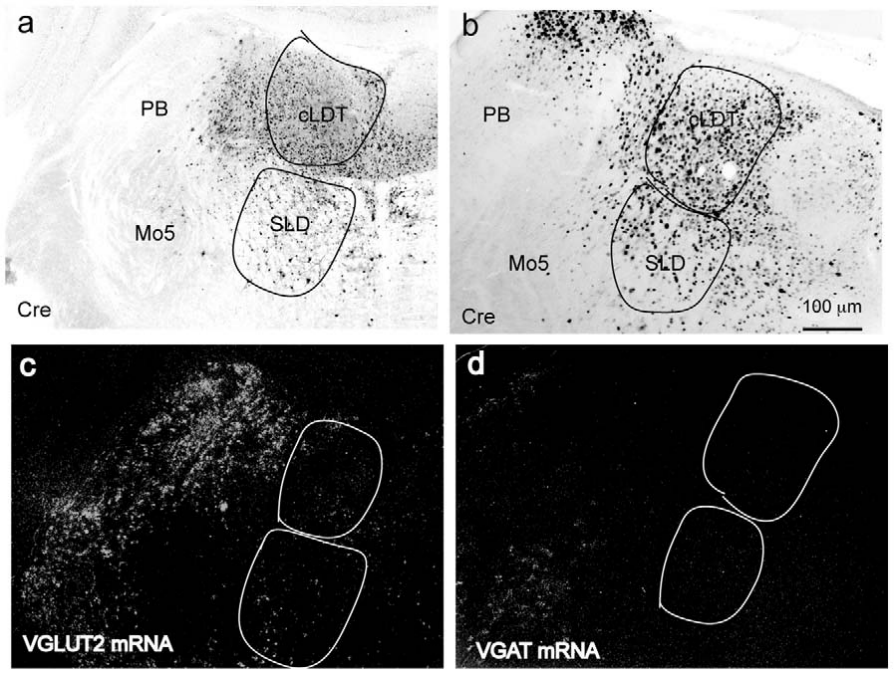
Verification of VGAT and VGLUT2 mRNA knockout in the cLDT-SLD. The white dots of “c” and “d” label VGLUT2 mRNA and VGAT mRNA in the cLDT-SLD respectively. The AAV-Cre injections in the cLDT-SLD region in a *Vglut2^flox/flox^* mouse (a) eliminate VGLUT2 mRNA (c) the glutamatergic PB that is not transfectionby AAV-Cre retains VGLUT2 mRNA. Similarly, AAV-Cre in the cLDT-SLD in a *Vgat^flox/flox^* mouse (b) eliminates VGAT mRNA (d).

Studies employing cell-body specific lesions in rats have suggested a critical role for the cLDT-SLD and the SLD alone in the control of REM sleep time and REM motor atonia, respectively [5]. We have further hypothesized that glutamate release from SLD neurons, in particular, is critical for motor atonia during REM sleep. Because, glutamate and GABA (and possibly other neurotransmitters and peptidergic systems) are intermingled in the cLDT-SLD (Figure 1b,c), it has been difficult to characterize the *in vivo* role of this fast neurotransmitter in regulating REM sleep phenomena, including REM motor atonia. Experiment 2 was thus performed using mice (n = 6) with loxP sites flanking exon-2 of the Vglut2 gene (*Vglut2^flox/flox^*). To selectively eliminate glutamate neurotransmission in SLD neurons, we injected AAV-Cre into the cLDT-SLD of the *Vglut2^flox/flox^* mice. As indicated above, when exposed to Cre recombinase, the second exon is deleted, and the gene is no longer capable of expressing functional protein and hence presynaptic release of glutamate from cLDT-SLD neurons was disrupted *in vivo* [13]. As vector-injection controls, we also injected AAV-GFP into the cLDT-SLD of six *Vglut2^flox/flox^* mice (Figure 1e and Figure 2).

**Figure 3:**
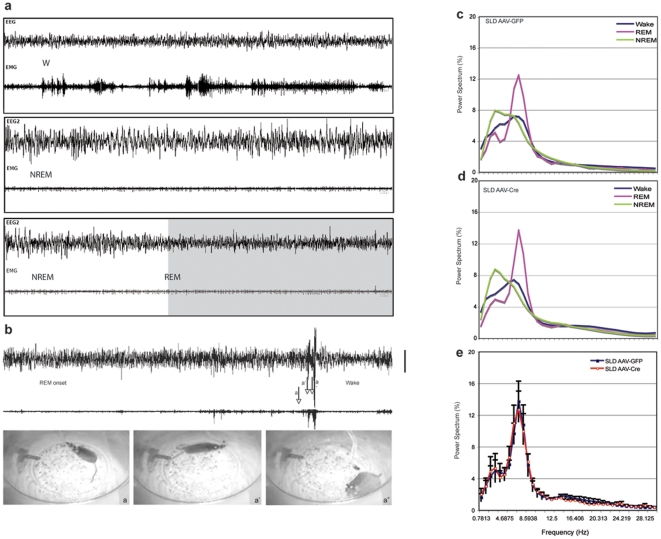
Normal sleep-wake and RBD like behavior episodes. Normal Wake, NREM and REM sleep EEG/EMG segments are shown in panel a. In panel b, a mouse with conditional VGLUT2 knockout in the cLDT-SLD exhibits normal NREM sleep and normal transition into REM sleep. Physiological atonia during REM sleep was observed only for a brief period which was followed by abnormally high muscle tone with kicking, jerking and running during REM episode. Three images within less than two seconds are captured marked by a, a' and a”. The tail, posture and animal positions indicate abrupt movement at the end of REM sleep. The entire REM episode lasts about 25 seconds. Notice that the figure shows only the end of REM sleep because animals with RBD show the most dramatic and phasic locomotion (very different from normal waking) at this moment that can be easily visualized by the pictures. The jerking and kicking during REM sleep that are never seen during wakefulness and NREM sleep are hard to visualize by images; however, the supplementary video captures these behaviors very well. Power spectrum of wake, NREM, REM sleep in VGLUT2 knockout is easily distinguishable (c), and power spectrum of REM sleep between control and VGLUT knockout is virtually identical (d). The calibration bar is 200 µV.

Verification of VGLUT2 deletion in the cLDT-SLD is shown in Figure 2. Of the six mice with AAV-Cre injections, five mice showed Cre covering the cLDT and SLD bilaterally (Figure 1h). One mouse had Cre labeling in the cLDT bilaterally but the SLD was not filled. In all five mice showing bilateral Cre in the cLDT-SLD, but not in AAV-GFP injected control mice or in the mouse with Cre labeling restricted to the cLDT, we observed a dramatic loss of motor atonia during REM sleep. Continuous motor atonia during REM sleep was never observed in mice with bilateral Cre in the cLDT-SLD, although atonia was occasionally present during the first 1–3 s after REM sleep onset. Instead, we observed overt motor behaviors during REM sleep in the AAV-Cre injected *Vglut2^flox/flox^* mice and these behaviors included both simple movements (e.g., whole body twitches, jerking and jumping; see supplementary Video S1) and, occasionally, more complex motor behaviors, such as locomotion. Although we did not measure the EMG of the limb muscles, the time-locked video capture clearly revealed the occurrence and pattern(s) of limb movements during REM sleep. Of note, and as shown in both Figure 3b and supplementary Video S1, the limb movements and motor behaviors observed in the AAV-Cre injected *Vglut2^flox/flox^* mice during putative REM sleep without atonia were distinct from movements seen during wakefulness. In fact, the aberrant motor behaviors observed during REM sleep were very similar to those we have previously seen in rats with cell-body specific lesions of the SLD and, also, resembled fundamental features of human RBD. In summary, these observations strongly suggest that glutamatergic neurons of the cLDT-SLD mediate REM sleep motor atonia.

**Figure 4:**
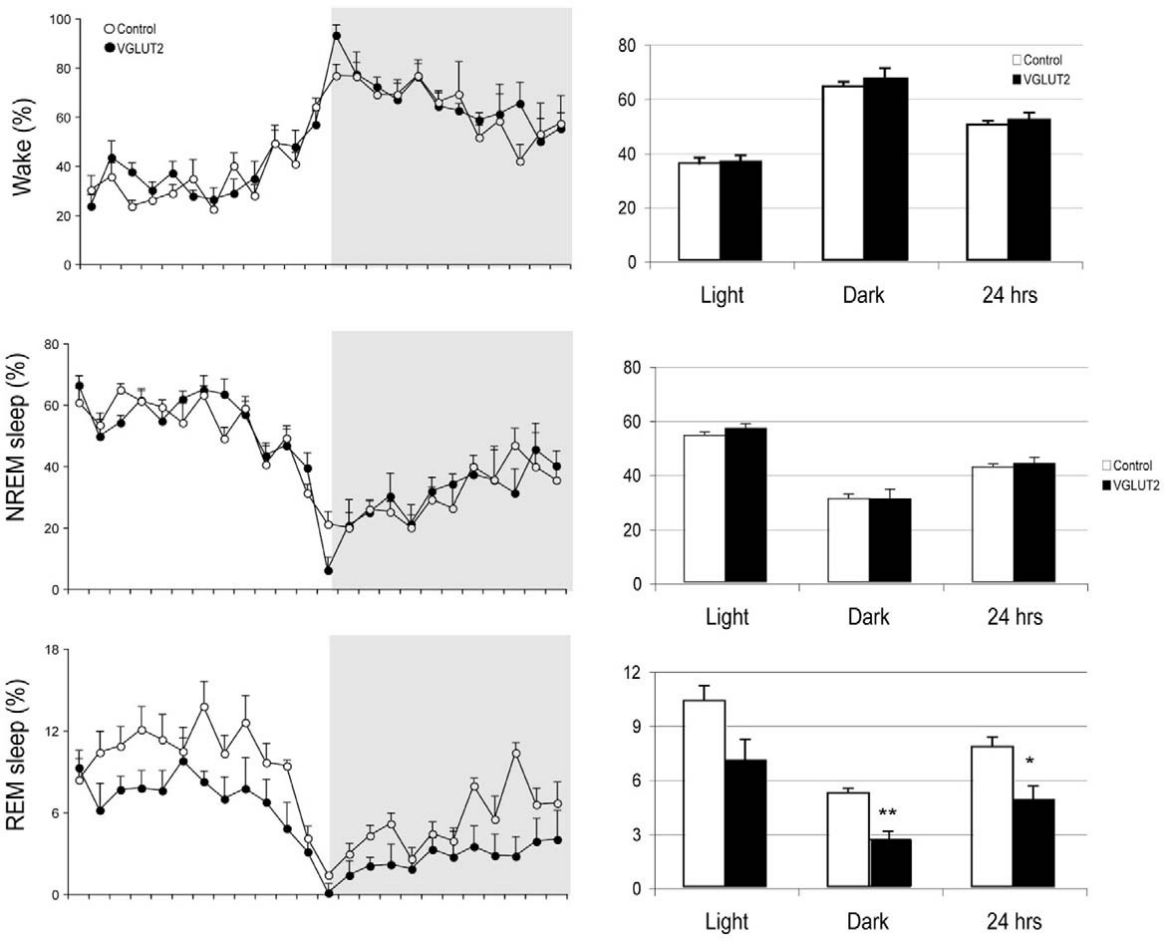
Circadian and day-night changes of wake, NREM sleep and REM sleep. The left panel shows an hourly percentage sleep-wake time over 24 hrs. The right panel shows the percentage of wake, NREM sleep, REM sleep time during light, dark and light+dark periods (24 hrs). REM sleep reduction in the VGLUT2 knockout group appears evenly across 24 hrs, although the magnitude is higher during the night. * p<0.05, ** p<0.01.

In addition to the absence of REM sleep motor atonia described above, we also observed a significant reduction in total REM sleep time in the AAV-Cre injected *Vglut2^flox/flox^* mice. Total (24 h) REM sleep time was decreased by nearly 40% in the AAV-Cre injected mice as compared to AAV-GFP injected mice (4.79±0.76% vs. 7.75±0.54%, p<0.05). The decrease in REM sleep time was most pronounced during the dark period (7 pm to 7am). In contrast to REM sleep time, neither the total amount of wakefulness nor NREM sleep was significantly altered in the AAV-Cre injected *Vglut2^flox/flox^* mice as compared with the AAV-GFP injected control mice. These findings therefore suggest that glutamatergic cLDT-SLD neurons might also play an important role in regulating REM sleep amounts (Figure 4).

**Figure 5:**
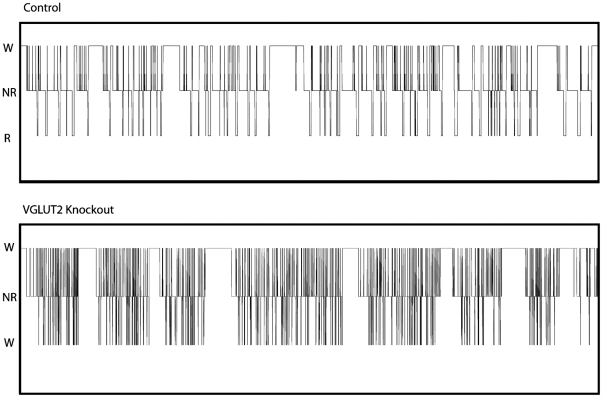
Stage changes of VGLUT2 knockout in the cLDT-SLD. Compared to a representative control animal (top), the mouse with VGLUT2 knockout (bottom) in the cLDT-SLD exhibits profound fragmentation in REM sleep, NREM sleep and wakefulness, and this is most evident during the light (rest) period (7:00–19:00).

Another interesting feature of the REM sleep state observed in the AAV-Cre injected mice was the severe fragmentation of REM sleep. REM sleep frequency, which we defined operationally as the number of REM episodes per 24 hours, was significantly increased by ca. 75% (162.3±22.6 versus 92.7±8.4 in the control group, p<0.01). A highly significant increase in frequency was also observed for NREM sleep (increase by 57%, 422.4±35.8 versus 269.8±26.1, p<0.01) and wakefulness (increase by 55%, 411.0±34.7 versus 265.2±26.6, p<0.01). The fragmentation of the sleep cycle was further reflected by a highly significant reduction in epoch duration during NREM (reduction by 34%) and REM (reduction by 65%). In summary, fragmentation of the sleep-wake cycle was most pronounced during the time that REM sleep was high, and REM sleep was more severely affected than NREM and wakefulness (Table 1, Figure 5).

**Table 1:** The effects of VGLUT2 and VGAT knockout on average bout duration (sec) and frequency of wake, NREM sleep and REM sleep across 24 hrs.

		WAKE		NREM		REM	
Genotype	Micro-injection	Average epoch duration	Frequency (epochs/24 hrs)	Average epoch duration	Frequency (epochs/24 hrs)	Average epoch duration	Frequency (epochs/24 hrs)
***Vglut2^flox/flox^***	**Control**	191.2±22.4	265.2±26.6	139.4±10.7	269.8±26.1	72.8±4.3	92.7±8.4
	**AAV-Cre into cLDT-SLD**	137.5±14.0	411±34.7**	92.6±10.3**	422.4±35.8**	25.4±3.5***	162.3±22.6*
***Vgat^flox/flox^***	**Control**	120.7±8.1	343.6±20.4	116.5±7.7	353.4±22.7	60.7±3.1	116.0±6.8
	**AAV-Cre into cLDT-SLD**	121.6±7.5	355.9±25.7	113.9±15.7	330.6±42.1	55.4±5.7	113.4±12.4
	**AAV-Cre into vlPAG-LPT**	116.8±8.8	309.8±16.8	140±12.1	330.1±17.5	68.3±2	95.9±6.8

*p<0.05,

**p<0.01,

***p<0.001.

### Experiment 4: Determine the role of GABAergic neurons in the REM-off and REM-on areas in REM sleep regulation

We have previously hypothesized that GABAergic neurons in the vlPAG-LPT may inhibit REM sleep via inhibition of REM-on neurons in the cLDT-SLD. Our model also predicts an important role for cLDT-SLD GABA in the regulation of REM sleep. For reasons similar to that for glutamate, however, it has proven difficult to characterize the *in vivo* role of GABA in regulating REM sleep phenomena. To approach this problem, we selectively disrupted GABAergic neurotransmission in the vlPAG-LPT using the same experimental approach as described in Experiment 2, but using the *Vgat^flox/flox^* instead of the *Vglut2^flox/flox^* mouse model. Similar in design to experiment 2, we selectively eliminated GABA neurotransmission in vlPAG-LPT or cLDT-SLD neurons by injecting AAV-Cre into the vlPAG-LPT or cLDT - SLD of the *Vgat^flox/flox^* mice (Figure 1h, Figure 2). As vector-injection controls, we also injected AAV-GFP into the vlPAG-LPT or cLDT-SLD of *Vgat^flox/flox^* mice (n = 5). Our results showed that the VGAT deletion in the cLDT-SLD or vlPAG-LPT did not result in any significant changes in REM atonia, REM sleep amounts or REM sleep architecture as compared with controls (Table 1).

### Experiment 3: Ascending projections of REM-on glutamatergic neurons

Given the above findings, we next sought to determine the potential pathways through which glutamatergic cLDT-SLD neurons might regulate the cortical features of REM sleep. With respect to putative structures involved in regulating the cortical features of REM sleep i.e., EEG desynchronization, we identified three candidate sites for injection of CTb: the basal forebrain, intralaminar thalamus and parabrachial nucleus (PB). We excluded the lateral hypothalamus as a candidate injection site because it receives only sparse afferent inputs from the cLDT/SLD region [14]. CTb was injected into one of each of these three sites in Vglut2-GFP mice (provided by Vong, L and Lowell BB, see methods). Glutamatergic projection neurons of the cLDT-SLD were then identified by double immunolabeling for CTb and GFP (n = 4 per site).

**Figure 6:**
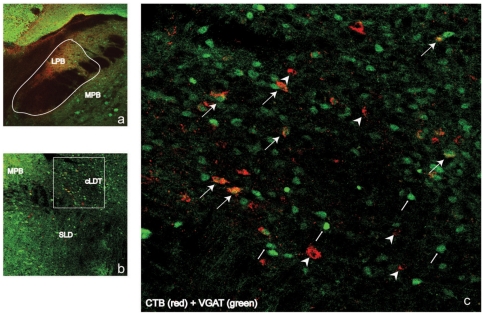
cLDT-SLD glutamatergic neurons project to the parabrachial nucleus. The retrograde tracer CTb injected into the parabrachial nucleus (mostly ventral LPB and MPB) bordered by a white line of VGLUT2-GFP mice (a) labels many neurons that are also glutamatergic in the cLDT-SLD (b, c). The box in (b) is enlarged in (c), where arrows indicate double-labeled neurons and arrowheads indicate single VGLUT2 labeled neurons.

Of the Vglut2-GFP mice receiving CTb injections only those with injections into the PB produced double-labeled cells in the cLDT-SLD (about 40% of CTb neurons were GFP+ and hence glutamatergic). This finding indicates that the PB receives glutamatergic inputs from the cLDT-SLD (Figure 6), whereas the basal forebrain and thalamus do not. The tracer data further suggests a potentially important role for the PB as an ascending “relay” for generating the cortical features of REM sleep. In contrast, we found no evidence that cLDT-SLD glutamatergic neurons project to the cortex via the thalamus or basal forebrain (supplementary Figures S1 and S2).

### Experiment 4. Spinal inhibitory interneurons mediate atonia

**Figure 7:**
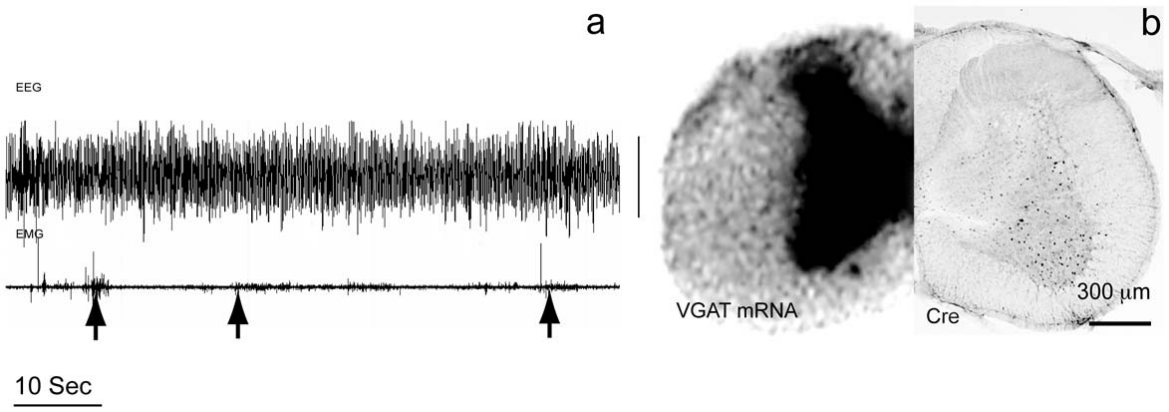
VGAT knockout in the spinal cord produces REM without atonia. “a” shows the absence of atonia (arrow points at twitches) during a REM sleep segment (high theta EEG) in a mouse with Vgat knockout in the C3–C4 ventral horn region (also see supplementary Video S2). “b” shows the Cre-labeled neurons (right side) in the ventral horn and loss of VGAT mRNA in the corresponding region (left). The calibration bar is 200 µV.

To determine whether inhibitory (GABA/glycine) interneurons in the spinal cord play a role in atonia, we placed bilateral injections of AAV-Cre (100 nl) into the C3–C4 ventral horn interneuron layer VII of *Vgat^flox/flox^* mice (n = 7). These mice were also outfitted with EEG/EMG recording leads. We found that mice with deletion of VGAT in the ventral horn region (n = 4) but not in the dorsal horn region (n = 3) at C3–C4 level exhibited twitching and jerking movements mostly in the upper and occasionally lower body extremities (including tail movements) during REM sleep (Figure 7, supplementary Video S2). We did not however observe any form of locomotion in the mice with loss of VGAT in the ventral horn during REM sleep. We did not observe detectable changes in motor behaviors during wakefulness and NREM sleep.

## Discussion

We have previously reported that cell-body specific lesions of the SLD (including unilateral lesions), but not the cLDT, in rats produced a loss of motor atonia during REM sleep. In addition, retrograde tracing studies have revealed that the SLD, but not cLDT, sends direct projections to both the ventromedial medulla (VMM) and ventral horn interneuron layers of the spinal cord, both of which are CNS regions implicated in REM atonia. Cell-body specific lesions of the entire cLDT-SLD, but not the cLDT or SLD alone, in rats produced a profound reduction in REM sleep (ca. 60%) and REM sleep fragmentation [5]. The present study provides the first *in vivo* evidence that glutamatergic neurons of the SLD play a critical role in the regulation of REM sleep and that these neurons together with glycinergic/GABAergic interneurons of the spinal ventral horn (and premotor neurons in the VMM) mediate REM atonia, whereas a presumably separate population of glutamatergic neurons in the cLDT and SLD are important for REM sleep consolidation. We also observed an unexpected absence of REM sleep changes following the *in vivo* disruption of GABA neurotransmission in the cLDT-SLD and the vlPAG-LPT regions.

Due to the relatively small size of the mouse SLD and cLDT, we did not attempt to distinguish between the effects of Vglut2 deletion in the SLD versus the cLDT. However, in one mouse loss of Vglut2 was restricted to the cLDT and although this mouse demonstrated a slight reduction in REM sleep (5.8%/24 hrs), there was no effect on REM sleep atonia. Taken together with results from previous rat lesion work [5], the present study suggests that SLD glutamatergic neurons projecting to the VMM and/or the spinal cord are involved in the regulation of REM sleep atonia, whereas a separate population of glutamatergic neurons [12], which are distributed over both the SLD and cLDT, are involved in the regulation of REM sleep timing and consolidation. We also demonstrated that glutamatergic cLDT-SLD neurons project directly to the parabrachial nucleus (PB), but not to the thalamus or basal forebrain, suggesting a possibly “relay” role for the PB in the regulation of the cortical and hippocampal EEG during REM sleep. For example, it has been shown previously that the PB sends excitatory glutamatergic projections to both basal forebrain corticopetal cholinergic and GABAergic neurons, which in turn regulate cortical and hippocampal activity [15]. Interestingly however we found that Vglut2 deletion in the cLDT-SLD did not alter the spectral characteristics of the REM sleep EEG, indicating that the glutamatergic cLDT-SLD neurons may not be the only neuronal population important for cortical activation during REM sleep.

The present study also suggests that GABA release from the vlPAG-LPT and cLDT-SLD is not critically involved in REM sleep control. This finding was unexpected on the basis of the results from several previous studies. For example, previous Fos studies have indicated that both GABAergic and glutamatergic neurons in the cLDT and SLD are REM-active [4], [5], [16]. It is also the case that cell-body lesions and injections of the GABA agonist muscimol into the vlPAG-LPT in rat [7], cat [9] and guinea pig increase REM sleep [10], and injections of GABA antagonists in the SLD trigger REM sleep in rats [4]. Finally, we had previously proposed a model in which mutual inhibitory projections from GABAergic neurons in the SLD and vlPAG-LPT may form the basis of REM sleep control. We found in the present study however that genetic disruption of GABA/glycine neurotransmission from these structures does not produce significant changes in REM sleep. While this finding is difficult to explain, we cannot exclude the possibilities that other, and as yet unrevealed, vesicular GABA transporters or co-localized inhibitory neurotransmitters in the GABAergic cLDT-SLD and vlPAG-LPT neurons may play a role in REM sleep control. It is also possible that GABAergic neurons in the cLDT-SLD might work synergistically with REM-controlling glutamatergic neurons (i.e., those identified in the present study) because the reduction in REM sleep observed following deletion of Vglut2 in the cLDT-SLD was smaller in magnitude than that produced by cell-body specific lesions in the same region in the rat.

As the cLDT also contains a small number of cholinergic neurons, the possibility exists that cholinergic neurons in the cLDT are also involved in REM sleep control. This idea has been previously proposed [17]. However, cell-body lesions of the cLDT alone do not affect REM sleep [5] and deletion of Vglut2 in the cLDT-SLD recapitulates the reduction in REM sleep seen following cell-body specific lesions of the same region. In addition, LDT cholinergic neurons do not contain glutamate or GABA [18]. Taken together, the available data support that the cLDT-SLD glutamatergic neurons regulate REM sleep.

As would be expected, REM sleep fragmentation resulted in overall changes in sleep-wake consolidation. We were however surprised at the magnitude of fragmentation in NREM sleep and wakefulness following Vglut2 deletion from cLDT-SLD. Despite the fragmentation, we did not observe a change in total NREM sleep and wake time. Similar patterns of fragmentation, albeit to a lesser degree, have also been reported in other animal models of disrupted sleep, including rats with lesions of the ventrolateral preoptic nucleus or mice with genetic disruption of orexin and/or histamine signaling [19], [20], [21].

Vglut2 deletion in the cLDT-SLD resulted in REM fragmentation characterized by reductions in REM sleep epoch duration coupled with an increase in the number of REM episodes (see Figure 5 and Table 1). These results indicate that glutamatergic neurons in the cLDT-SLD region are critical for the maintenance of REM sleep. Importantly, however, while neurons of the cLDT-SLD are clearly critical for generating and maintaining REM sleep, the regulation of REM sleep likely requires the participation of other brainstem structures, such as circuitry in the ventromedial medulla [12]. For example, it has been shown that pretrigeminal transections in the cat virtually eliminated REM sleep [22] and that transections caudal to the cLDT-SLD blocked REM induction by carbachol injection into the pons in cats [23]. These findings strongly suggest that cLDT-SLD neurons and REM-active medullary circuits work together to generate and maintain REM sleep.

Chase and colleagues first showed that glycine mediates motor inhibition during REM sleep, however the source of this inhibition has been long debated. We have previously hypothesized that spinal cord inhibitory premotor neurons are critical for REM atonia [5], [12]. In the present study we found that deletion of GABA/glycine release from spinal ventral horn interneurons resulted in deficits in REM atonia in mice. As GABA/glycine deletion was confined to C3–C4 in our experiments, it was not surprising that the magnitude of disinhibition of the motor behaviors was much less than that caused by SLD glutamate deletion. Ultimately, it will be necessary to produce deletion of spinal glycine/GABA transmission along the entire spinal axis to demonstrate that inhibitory premotor neurons in the spinal cord are responsible for REM sleep atonia. This was not possible in the present study.

We have previously proposed that glutamate in the VMM might be critical for suppressing phasic muscle activity during REM sleep as disruption of glutamate transmission in the ventral medulla produces more prominent phasic activity of EMG than disruption of GABA/glycine transmission. Importantly, however, neither glutamate nor GABA/glycine deletion from the VMM nor VMM lesions recapitulated the complete loss of muscle atonia or the overt motor behaviors during REM sleep as seen in the SLD Vlgut2 knockout [12]. Thus, glutamatergic neurons in the SLD may act on all three populations (glutamatergic and GABA/glycinergic neurons in the VMM and GABA/glycinergic interneurons in the spinal cord) to bring about muscle atonia during REM sleep. In addition, simultaneous withdrawal of monoaminergic and orexinergic neurotransmission may also be required for generation and maintenance of muscle atonia as previously suggested [12], [24].

The loss of REM atonia observed following genetic deletion of Vglut2 from the SLD was highly reminiscent of human RBD. RBD is a parasomnia typically manifested as ‘dream enactment’ behavior' and is thought to represent an early pathophysiologic manifestation of evolving Parkinson's disease (PD) and other synucleinopathies, e.g., Lewy body dementia (LBD), multiple system atrophy (MSA) and pure autonomic failure [25], [26], [27]. Mathis and colleagues recently reported on a case of RBD that developed following a rare encephalitis-induced lesion that was restricted to the dorsal pontine tegmentum and presumably involved the subcoeruleus region bilaterally. Also interesting is the recent report of human RBD development following a stroke in the right SLD region [28]. Finally, a recent imaging study has reported tissue abnormalities in the pontine tegmentum in an idiopathic RBD case [29].

## Methods

### Animals

The male mice were housed in the Center for Life Science animal facility and the care of the animals in this study met National Institutes of Health standards, as set forth in the *Guide for the Care and Use of Laboratory Animals*. All protocols were approved by the Beth Israel Deaconess Medical Center Institutional Animal Care and Use Committee (Animal Welfare Assurance #A3153-01). The animals were individually housed in standard plastic mouse cages with *ad libitum* access to food (Lab Diet) and water. The cages were housed inside isolation chambers, which provided ventilation, computer-controlled lighting (12:12 light-dark cycle, lights on at 07:00; 200 lux), an ambient temperature of 22±1°C, and visual isolation.

#### Conditional Knock-out Mice

The generation and characterization of the *Vgat^flox/flox^* and *Vglut2^flox/flox^* mice were described in detail previously [13], [30].

#### VGLUT2-GFP and VGAT-GFP mice

These mice are generated and provided by Drs. Vong and Lowell. To generate the VGLUT2-GFP and VGAT-GFP mice used in Experiment 4, Vglut2-ires-cre and Vgat-ires-cre mice were bred to lox-GFP reporter mice (cite paper). As expected, Cre activity as measured by GFP immunohistochemistry was observed only in glutamatergic and GABAergic neurons, respectively.

### Tracer injections

Tracer injections into the brain and spinal cord have been described in detail previously [5], [12], [17].

#### Microinjection and implantation of EEG/EMG leads

To selectively eliminate glutamate and GABA/glycine neurotransmission, we injected 100 nl of an adeno-associated viral (AAV) vector containing Cre recombinase (Cre) into the cLDT-SLD (coordinates: AP = −5.3 mm, ML = ±0.7 mm, DV = −3.4 mm) or vlPAG-LPT (coordinates: AP = −4.2 mm, ML = ±0.8 mm, DV = −3.0 mm) of the *Vglut2^flox/flox^* and *Vgat^flox/flox^* mice (25–35 g). AAV-GFP was injected into different *Vglut2^flox/flox^* or *Vgat^flox/flox^* mice to serve as controls. All injections were performed using the same compressed air delivery system previously described [21]. Immediately following injections of either AAV-Cre (Serotype 10, 90 nl) or AAV-GFP (Serotype 10, 90 nl), four EEG electrodes (0.1″, Pinnacle Technology Inc.) and two nuchal EMG electrodes (Plastics One), previously soldered to a 6-pin connector (Pinnacle Technology Inc.), were implanted (AP+1.0 mm/RL ±1 and AP −2.0 mm/RL ±1.0 mm) and insulated with dental cement [31].

Similarly, for anatomical tracing experiments, a retrograde tracer, cholera toxin subunit b (CTb) was injected into the basal forebrain (AP = −0.1 mm, ML = ±1.5 mm, DV = −4.2 mm), intralaminar thalamus (AP = −1.0 mm, ML = ±0.5 mm, DV = −3.0 mm) or parabrachial nucleus (AP = −5.3 mm, ML = ±1.3 mm, DV = −2.3 mm) in VGLUT2-GFP mice. These animals were not implanted with EEG/EMG electrodes.

#### EEG/EMG recording and sleep-wake analysis

Three weeks after surgery, the mice were connected via flexible recording cables, containing a preamplifier, to a commutator, which in turn was connected to an amplifier (Pinnacle Technology Inc.) and a computer. The mice were habituated to the setting for three days. Continuous recording of the EEG/EMG and time-lock video (Sirenia Pinnacle Technology Inc.) began after the habituation period and continued for 48 hours. Three behavioral states (Wake, NREM and REM sleep) were visually identified and analyzed in 10-s epochs using SleepSign for Animal (Kissei, Japan) [12].

The percentage of time spent in wake, NREM sleep and REM sleep, frequency and the average durations of each stage were summarized for each group. Statistical differences in sleep-wake time, episode number, and duration were assessed using a Student's two-tailed t-test, after a normal distribution was confirmed for each group and a test for variance homoscedasticity was performed. An α<0.05 was considered significant.

For the EEG power spectrum analysis, 4 hrs (7–11pm) of spontaneous recordings were scored again in 10 s epochs. On the basis of visual and spectral analysis, epochs containing artifacts occurring during active wake (with large movements) or immediately before or after other vigilance states were visually identified and omitted from the spectral analysis. EEG power spectra were computed for consecutive 10 s epochs within the frequency range of 0.5–60 Hz using a fast Fourier transform routine. The data were collapsed in 0.75 Hz bins. The power densities obtained for each state were summed over the frequency band of 0.5–60 Hz (total power). To standardize the data, each frequency bin was expressed as a percentage relative to the total power (e.g., bin power/total power) of the same epochs. To analyze the EEG frequency bands, relative power bins were summed in δ 0.5–2.5 Hz, slow θ 2.5–5.5 Hz, fast θ 5.5–10, α 10–20 Hz, β+γ 20–60 Hz.

#### In situ hybridization and immunohistochemistry

Immunohistochemical staining for Cre and GFP was performed as previously described previously [12], [31]. In situ hybridization for labeling of Vglut2 mRNA or Vgat mRNA was also performed as previously described and is also illustrated by Figure 2 [5], [12], [17], [31], [32].

## Supporting Information

Video S1 **RBD-like behavior after genetic disruption of Vglut2 in the mouse SLD-cLDT.** Almost every REM episode following genetic disruption of glutamatergic transmission in the cLDT-SLD [by injections of AAV-Cre in a *Vglut2^flox/flox^* mouse] was marked by an absence of atonia, and which ranged from simple movements (e.g., whole body twitches, jerking and jumping) to, occasionally, more complex motor behaviors (e.g., locomotion).
(MP4)

Video S2 **REM sleep without atonia after conditional knockout of Vgat in the spinal cord.** Injection of AAV-Cre into the C3–C4 interneuron level of a *Vgat^flox/flox^* mouse resulted in an absence of atonia during many REM episodes and this was characterized by twitches, esp. in the cervical region and upper body but also occasionally in the lower body (legs and tail).
(AVI)

Figure S1 **cLDT-SLD glutamatergic (VGLUT2) neurons do not project to the thalamus.** Although CTb injected into the intralaminar thalamus (a) retrogradely labels numerous large-size neurons (red color) in the cLDT, most of these cells are likely cholinergic (b), and CTb-labeled neurons in the cLDT-SLD rarely contained VGLUT2 (green color) (b). Arrows indicate double-labeled neurons.
(TIF)

Figure S2 **cLDT-SLD glutamatergic (VGLUT2) neurons do not project to the basal forebrain.** CTb injected into the basal forebrain (a) retrogradely labels many glutamatergic neurons in the parabrachial nucleus (PB) but rarely glutamatergic neurons in the cLDT-SLD. Arrow indicates a double-labeled neuron (yellow color); lat PB: lateral parabrachial nucleus; med PB: medial parabrachial nucleus; MDT: mediodorsal thalamus; scp: superior cerebellar peduncle; ven: fourth ventricle.
(TIF)
