# Supplementary figures and images for: Brainstem and Spinal Cord Circuitry Regulating REM Sleep and Muscle Atonia
Source: PLoS One. 2011 Oct 17;6(10):e24998. doi: 10.1371/journal.pone.0024998 (PMC3197189; doi:10.1371/journal.pone.0024998)

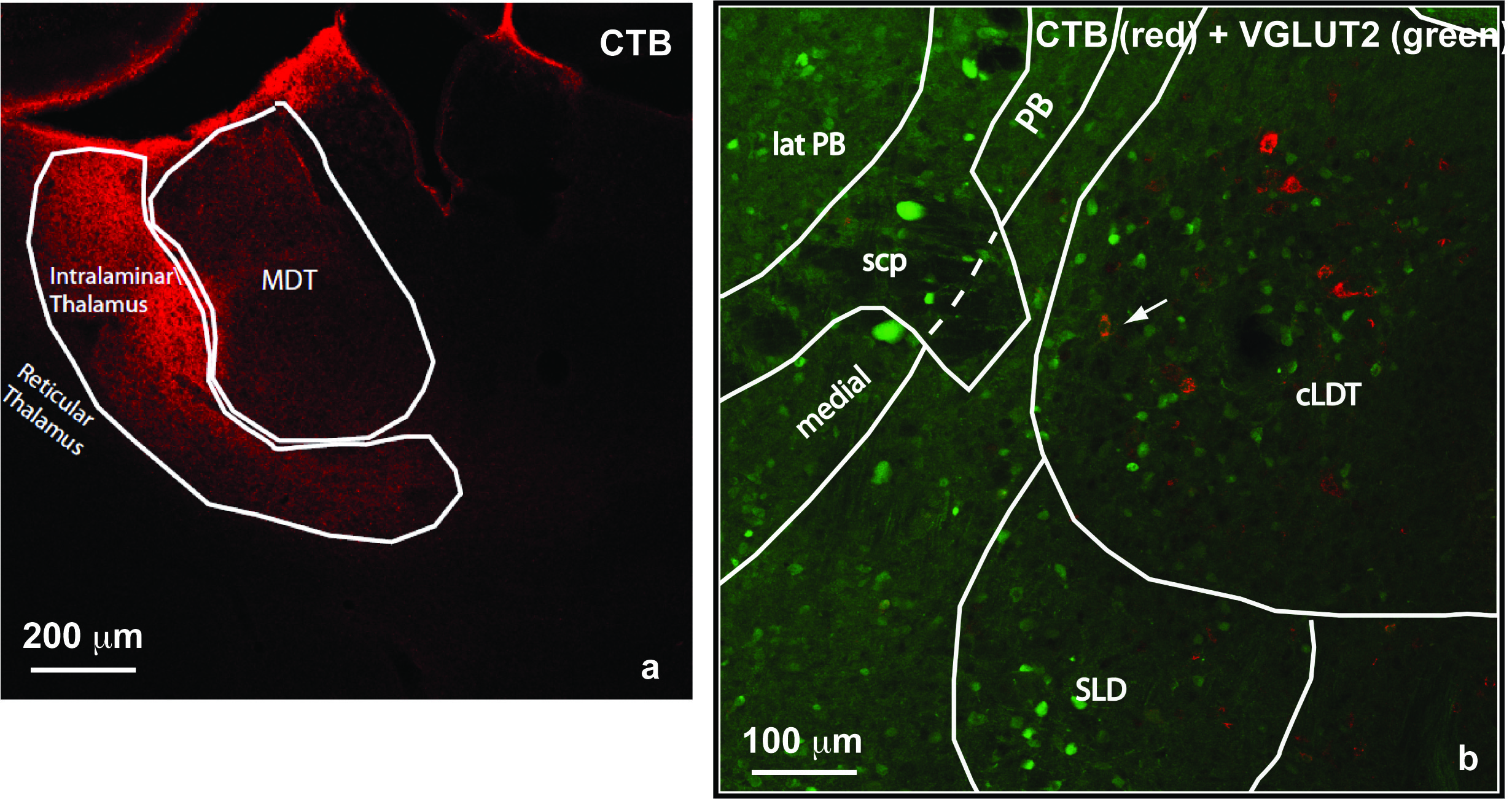

Supplement: Figure S1 — cLDT-SLD glutamatergic (VGLUT2) neurons do not project to the thalamus. Although CTb injected into the intralaminar thalamus (a) retrogradely labels numerous large-size neurons (red color) in the cLDT, most of these cells are likely cholinergic (b), and CTb-labeled neurons in the cLDT-SLD rarely contained VGLUT2 (green color) (b). Arrows indicate double-labeled neurons. (TIF) [file pone.0024998.s003.tif]

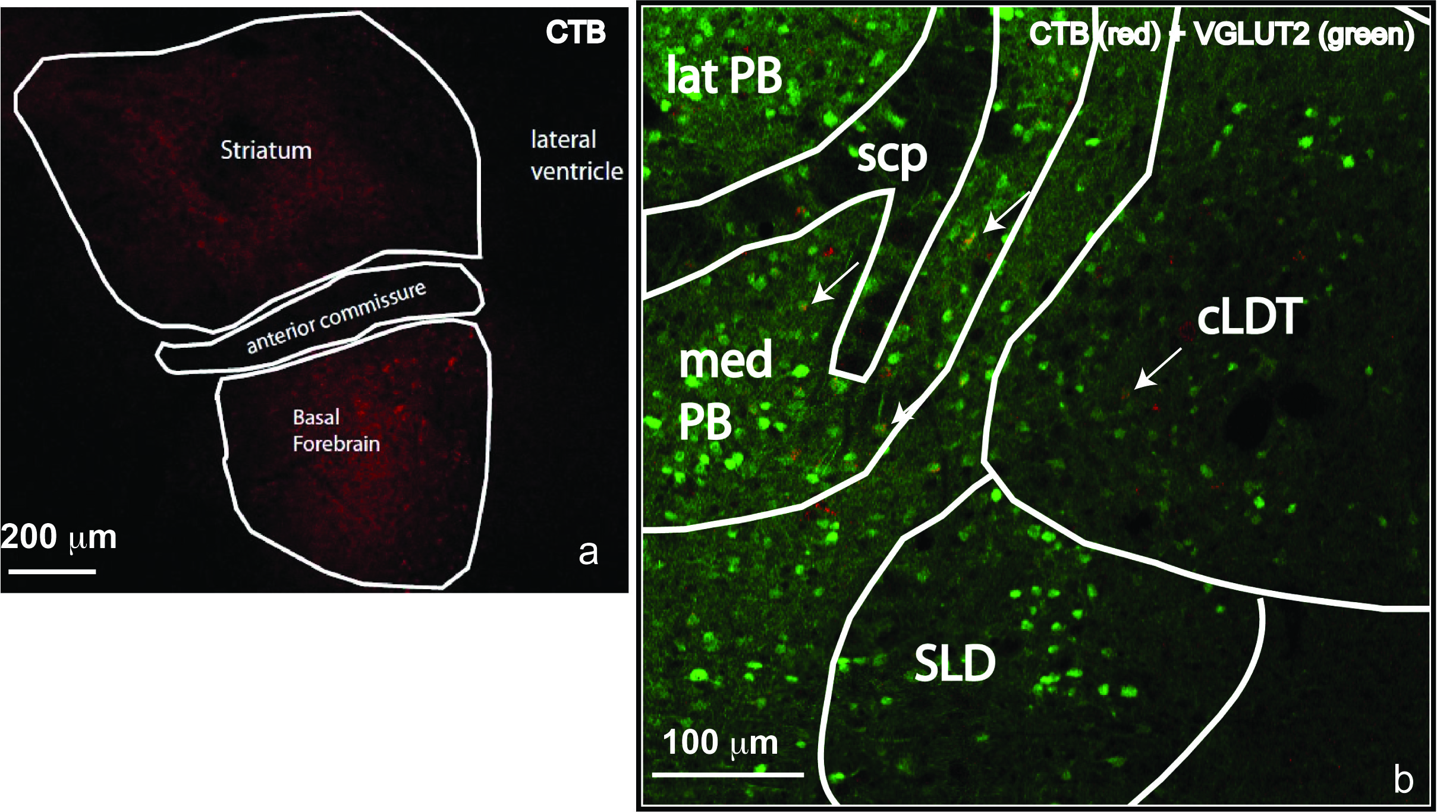

Supplement: Figure S2 — cLDT-SLD glutamatergic (VGLUT2) neurons do not project to the basal forebrain. CTb injected into the basal forebrain (a) retrogradely labels many glutamatergic neurons in the parabrachial nucleus (PB) but rarely glutamatergic neurons in the cLDT-SLD. Arrow indicates a double-labeled neuron (yellow color); lat PB: lateral parabrachial nucleus; med PB: medial parabrachial nucleus; MDT: mediodorsal thalamus; scp: superior cerebellar peduncle; ven: fourth ventricle. (TIF) [file pone.0024998.s004.tif]
